# Carbon footprint of the Chinese healthcare service: An environmentally extended input–output analysis

**DOI:** 10.1371/journal.pmed.1004738

**Published:** 2025-09-24

**Authors:** Juan Liang, Rui Wu, Peng Bi, Shi-Lu Tong, Rui Zhang, Xiao-Yuan Yao, Xin Jin, Yong-Hong Li

**Affiliations:** 1 China CDC Key Laboratory of Environment and Population Health, National Institute of Environmental Health, Chinese Center for Disease Control and Prevention, Beijing, China; 2 School of Business, Nanjing Normal University, Nanjing, China; 3 School of Public Health, The University of Adelaide, Adelaide, Australia; 4 School of Public Health and Social Work, Queensland University of Technology, Brisbane, Queensland, Australia; 5 School of Public Health, Institute of Environment and Population Health, Anhui Medical University, Hefei, China; 6 Chinese Center for Disease Control and Prevention, Beijing, China; Washington University in St. Louis, UNITED STATES OF AMERICA

## Abstract

**Background:**

With the healthcare sector contributing nearly 5% of total global greenhouse gas (GHG) emissions globally, a precise assessment of their carbon footprint is crucial for achieving carbon neutrality targets. This study aims to comprehensively assess the carbon footprint of Chinese healthcare service providers, to identify their driving activities and sources across different time periods, and to provide a solid foundation for the development of effective emission reduction policies in healthcare service in China.

**Methods and findings:**

The data on overall national health expenditures for 2012 and 2018, as well as expenditures by different levels of hospitals, various hospital departments, and specific diseases, were sourced from China’s Health Statistics Yearbooks and national input–output tables (IOTs). Environmentally extended input–output analysis (EEIOA) and structural path analysis (SPA) were utilized to assess the carbon footprint of healthcare services in China in 2012 and 2018. Overall, the total carbon footprint of Chinese healthcare service providers increased by 51 MtCO_2_e (15%) in 2018 compared to that in 2012, accounting for about 3.7% of the total domestic GHG emissions. In 2018, public hospitals made the largest contribution to the carbon footprint within the national health expenditure categories, with their carbon emissions increasing by 29 MtCO_2_e (19%). Among medical institutions, procurement was the largest contributor to the carbon footprint, with emissions increasing by 46 MtCO_2_e (25%). Within hospital departments, the internal medicine department had the highest carbon footprint, reaching 47.66 MtCO_2_e (26%) in 2018. When classified by hospital grades, tertiary hospitals contributed the most, emitting 126.50 MtCO_2_e (70%). When classified by disease category, circulatory system diseases had the largest carbon footprint of 12.68 MtCO_2_e (19%), while malignant neoplasms were the primary contributor among subcategory diseases, emitting 5.52 MtCO_2_e (8%). The main limitation of this study lies in the fact that national IOTs are updated approximately every 5 years, and data for methane (CH₄) and nitrous oxide (N₂O) have not been updated since 2018. As a result, the analysis could only be performed for the years 2012 and 2018.

**Conclusions:**

These findings highlighted the substantial GHG emission contributions in China from public hospitals, especially tertiary hospitals, procurement activities, Internal Medicine Departments, and specific diseases in the carbon footprint. The findings provided robust scientific evidence for formulating strategies to reduce carbon emissions within the healthcare service in China and will also have implications for other countries.

## Author summary

**Why was this study done?:**

- We found that the last study assessing the carbon footprint of Chinese healthcare was done a decade ago and only focused on the expenditure category level.
- Here, we provide an updated analysis, which also incorporated other factors, including an aging population, increasing healthcare demands, new medical infrastructures and facilities, and their contributions to carbon emissions.

**What did the researchers do and find?:**

- We used correlation analysis of economic system and environmental data to track energy and material flows in the medical supply chain to quantify the carbon emission dynamics of China’s healthcare service.
- We further assessed public hospitals from three perspectives, including hospital level, departmental composition, and disease type.
- We found that the total carbon footprint of Chinese healthcare service providers increased by 51 MtCO_2_e (15%) in 2018 compared to that in 2012. Tertiary hospitals, internal medicine departments and specific diseases (circulatory diseases, tumors, injuries, and poisonings, etc.) contributed significantly to GHG emissions in public hospitals.

**What do these findings mean?:**

- Healthcare sector can contribute significantly to global climate governance and sustainable development goals by maintaining high-quality medical services while effectively controlling carbon emissions.
- The data in this study have a lag. The national input–output table is updated every 5 years, and the CH₄ and N₂O data have not been updated, so calculations can only be made up to 2018.

## Abstract 3

In an environmentally extended input-output analysis, Juan Liang and colleagues performed correlation analysis of economic system and environmental data to assess the carbon footprint of the Chinese health care system.

## Introduction

Climate change poses a dual challenge to healthcare systems worldwide [1,2]. In many parts of the world, the cumulative impacts of climate change are placing an even greater burden on an already strained healthcare system [3]. Rising temperatures and extreme weather events such as heatwaves [4], typhoons [5], floods [6], and droughts [7] increase the burden of respiratory, cardiovascular, and infectious diseases [8,9]. Additionally, climate change indirectly exacerbates the risks of malnutrition and waterborne diseases by affecting food security and water supply [10], further straining already overstretched healthcare infrastructure. Simultaneously, the healthcare sector itself is a significant contributor to global greenhouse gas (GHG) emissions, accounting for about 5% of the total, with high-income nations responsible for 3%–10% of their national carbon footprints [11]. These emissions stem not only from direct sources (e.g., hospital energy use) but also from indirect supply chain activities (e.g., the procurement of pharmaceuticals, consumables, medical equipment, and services). Given the sector’s rapid expenditure growth driven by aging populations, chronic diseases, and technological advances [12], addressing its carbon footprint is critical to aligning healthcare delivery with global climate goals [13].

Carbon footprint as an important indicator of GHG emissions, has been utilized globally to assess climate change threats [14]. The definition of carbon footprint refers to the amount of GHG emissions, both direct and indirect, produced by an entity (such as a product, service, organization, or individual) throughout its entire life cycle [11]. It encompasses not only carbon dioxide emissions but also emissions of other GHGs (such as methane, nitrous oxides, etc.), typically expressed in terms of carbon dioxide equivalent (CO_2_e) to provide a comprehensive representation [15].

Evidence shows healthcare expenditures have grown faster than economic growth in recent decades, driven by population aging, lifestyle-related noncommunicable diseases, and rapid medical advancements [12]. Research on healthcare carbon footprints reveals significant cross-national variations due to differing policies [16]. For example, the carbon footprint of the healthcare system in the United States reached 655 MtCO_2_e in 2013, accounting for 10% of the nation’s total GHG emissions [17], while Canada’s was 33 MtCO_2_e (4.6% of the national emissions) in 2014 [18]. Other countries showed similar patterns: Australia (36 MtCO_2_e, 7% during 2014 and 2015) [19], Austria (6.8 MtCO_2_e, 7% in 2014) [20], Japan (72 MtCO_2_e, 5.2% in 2015) [21], and the UK (27 MtCO_2_e, 4% in 2019) [22]. These findings demonstrate that healthcare systems are major contributors to national GHG emissions.

However, most current carbon footprint calculations in healthcare services were undertaken in developed countries, with limited research from developing countries. As the largest developing country, China ranks the second-largest carbon footprint in the global healthcare service with 17% of GHG emissions [23]. Our preliminary assessment of the carbon footprint of Chinese healthcare services found that, by expenditure category in 2012, it emitted 315 MtCO_2_e, accounting for 2.7% of the total domestic GHG emissions in China [15]. Given this study only focused on expenditure category level, and was undertaken a decade ago, it is necessary to undertake an updated analysis in the Chinese healthcare services to incorporate other factors, including aging population, increasing healthcare demands, new medical infrastructures and facilities, and their contributions to carbon emissions to inform the development of effective strategies for GHG reductions.

To fill these research gaps, the study aims to examine the carbon footprint of the healthcare service in China, analyze its carbon emission hotpots and structural pathways from 2012 to 2018, and further conduct a classified study on the largest emission category in public hospitals. The findings could provide a more precise estimation of carbon footprint in healthcare services in China, offer evidence for the implementation of effective measures to reduce carbon emissions for green and low-carbon transition.

## Methods

In this study, the environmentally extended input–output analysis (EEIOA) was used [24], which adopts a “top-down” model and is usually applied to macro-level carbon footprint calculations, such as for countries, departments, or enterprises. Compared with other methods [25,26], EEIOA is generally based on the Leontief inverse matrix of the value-based input–output model. It captures the environmental pressures that are directly and indirectly caused by the production activities of the various sectors, considering the supply chain pathways among all the production sectors within the healthcare system. The strength of this method lies in its ability to comprehensively assess carbon footprints without requiring system boundary selection, while simultaneously overcoming the “truncation error” problem. It is worth noting that there are some limitations in the application of the EEIOA method, as its reliance on historical input and output data leads to a time lag in the analysis results. In addition, the EEIOA method is a suitable tool for assessing the macro carbon footprint of healthcare services and should be supplemented with other methods when formulating emission reduction plans for specific healthcare institutions. This study is reported as per the Strengthening the Consolidated Health Economic Evaluation Reporting Standards 2022 (CHEERS 2022) statement (S1 CHEERS Checklist).

### Data collection

All data was collected from publicly available datasets. The national Input–Output Tables (IOTs) for 2012 and 2018 were collected from the China National Bureau of Statistics (NBS) (https://www.stats.gov.cn/). The IOTs were constructed using a series of official statistical and national economic accounting data. The national CO_2_ emissions inventory for 2012 and 2018 was obtained from the China Carbon Accounting Databases (CEADs) (https://www.ceads.net.cn/), and the CH_4_ and N_2_O emissions inventories were obtained from the National Communication on Climate Change of the People’s Republic of China (https://www.mee.gov.cn/). Data on health expenditures for 2012 and 2018 were originated from the National IOT (https://www.stats.gov.cn/), China Health Statistics Yearbook (https://www.nhc.gov.cn/), China Construction Statistics Yearbook (https://www.stats.gov.cn/), and China Science and Technology Statistics Yearbook (https://www.stats.gov.cn/). Expenditures data for hospitals across various levels, individual departments, and specific diseases in 2018 were sourced from China Health Statistics Yearbook (https://www.nhc.gov.cn/).

### Data analysis

A two-stage approach was adopted to assess the carbon footprint of healthcare services in China. Stage 1: The carbon footprint of the whole life cycle of China’s healthcare system was quantified using Environmental EEIOA [27]. Initially, according to the standard of the China Industrial Classification for National Economic Activities (GB/T 44,754-2017), the national IOT was divided into 45 economic sectors. Subsequently, a GHG emissions account was constructed for each of these economic sectors. The total GHG emissions of a sector were divided by its corresponding monetary output to obtain the direct carbon emission intensities for that sector. Thereafter, the direct carbon emission intensities, which represent the emissions directly associated with each economic sector’s production, were multiplied by the Leontief inverse matrix to obtain the total carbon emission intensities at basic economic prices. Finally, the total carbon emission intensities were multiplied by the health expenditure data to obtain the carbon footprint of the healthcare service. Additionally, to calculate the carbon footprint of hospitals of different levels and individual departments within the hospital, the relevant expenditures were multiplied by the total emission factor of the medical institutions. Similarly, to calculate the carbon footprint of specific diseases, the relevant expenditures were multiplied by the weighted average of the total emission factors of the medical institutions and nonhospital purchased (NHP) pharmaceuticals. Stage 2: Structural path analysis (SPA) was utilized to decompose the healthcare institutions [28]. The first level of decomposition involves direct emissions from medical institutions, the second level focuses on indirect emissions from the procurement of medical supplies and services, and the third level decomposes the indirect emissions from the upstream procurement of pharmaceuticals within the supply chain. This approach not only identifies the key drivers of the healthcare system but also reveals how these factors influence the outcome through different pathways, thus providing a theoretical basis for policy making and system optimization.

The IOTs for 2012 and 2018 were divided into 45 economic sectors, respectively [15]. The GHG emissions for each sector, expressed in carbon dioxide equivalents, were obtained by multiplying the emissions of CH_4_ and N_2_O by their 100-year global warming potential (GWP) [15]. The healthcare expenditures were categorized into nine categories based on the China Health Statistics Yearbook [15], which are medical institutions (including public hospitals, private hospitals, community healthcare services, public health services, and other healthcare institutions), NHP pharmaceuticals, construction, management, and research (Table 1). Each hospital is divided into 22 departments, categorized based on outpatient and inpatient services. The hospitals are classified as primary, secondary, and tertiary hospitals. Diseases classified under the 10th revision of International Classification of Diseases (ICD-10) comprise 20 major categories and 154 subcategories, and are further categorized by city and county, gender, and age.

**Table 1:** Detailed health expenditure description of Chinese Healthcare Service. Abbreviation: NHP, nonhospital purchase.

Expenditure categories	Description
Public hospitals	State-owned and collective hospitals.
Private hospitals	Hospitals outside of state-owned and collective.
Community healthcare	Including community health service centers, subdistrict health stations, township health centers, village clinics, and private medical practices (clinics).
Public health	Including disease control and prevention centers, specialized disease control institutions, maternal and child healthcare institutions, health education institutions, emergency medical centers, blood collection and supply stations, health supervision institutions, and family planning technical service institutions.
Other healthcare institutions	Including sanatoriums, clinical testing centers, medical research institutions, medical continuing education institutions, medical examination centers, talent exchange centers, statistical information centers, and other health institutions.
NHP pharmaceuticals	Exclusively including noncommunicable disease medications sold to consumers in pharmacies.
Construction	Including hospital construction and maintenance.
Management	All administrative activities within government health departments (excluding administrative activities within hospitals)
Research	Medical research (including research institutes and universities).

Note: Compiled from the China Yearbook of Health Statistics.

### Input–output analysis

This study employs input–output analysis to calculate the direct and total GHG of Chinese Healthcare Service. According to the standard input–output equation [15], the relationship is as follows:


X=Z+f (1)

In (Eq. 1), $X=x_{i}$ represents the column vector of total output across various economic sectors. $Z=z_{ij}$ denotes the direct consumption of products from sector i by sector j. $f=f_{i}\;$indicates the column vector of the i sector’s products as final demand. The transformation from (Eq. 1) to (Eq. 2) is expressed as follows:


$$X=(I-A{)}^{-\text{1}}f=Lf$$ (2)

(Eq. 2) represents the Leontief model, which is the most core and crucial formula in input–output technology. It reflects the relationship between final demand and total output. $L=(I-A{)}^{-\text{1}}\;$ is known as the Leontief inverse matrix, which comprehensively reveals the intricate economic interdependence among various sectors of the national economy. Where I is the unit matrix, $A=a_{ij}$ is the direct consumption coefficient matrix, also written as $A=Z{\hat{X}}^{-\text{1}}$. Z is the intermediate transaction matrix, with the ^ symbol indicating a diagonal matrix where the vector elements are along the diagonal, and other elements are zero. The calculation method for the A matrix involves dividing each element of the *Z* matrix by the total output of the corresponding sector, $a_{ij}=\frac{z_{ij}}{X_{j}}$, where $a_{ij}$ represents the direct consumption coefficient of the i sector’s products by the j sector to produce one unit of its output. According to the input–output balance equation, $x_{j}=x_{i}$. The row vector for calculating the carbon footprint of the health sector(C)is given by the relationship:


C=M\Hatf (3)

In (Eq. 3), $M=m_{i}$ represents the total emission intensity of the i sector, which can be obtained by multiplying the direct emission intensity row vector D by the Leontief inverse matrix L. $D=d_{i}$ represents the direct emission intensity of the i sector, which can be calculated by dividing the GHG emissions E by the total output X of the corresponding sector. The direct emission intensity row vector D and the total emission intensity column vector D correspond to the column vectors in the intermediate transaction matrix Z.

### Structural path analysis

Structural path analysis (SPA), first proposed by Defourny and colleagues in 1984, decomposes the total emissions of an economy into an infinite number of paths within its production system, ranking these paths by their direct emission contributions. An economic system comprises multiple factors, where a change in one factor leads to corresponding changes in other interconnected factors, which in turn affect further factors, and so on and so forth, layer upon layer.


$$(I-A{)}^{-\text{1}}=I+A+A^{\text{2}}+A^{\text{3}}+\cdots$$ (4)

Therefore, the carbon footprint $C_{m}$ of the medical institution sector can be divided into direct emissions$\;{\;C}_{m}^{\text{0}}$, first-level indirect emissions ${\;C}_{m}^{\text{1}}$, and all subsequent indirect emissions ${\;C}_{m}^{\text{2}n}$, and the relationship is:


$$C_{m}={\;C}_{m}^{\text{0}}+{\;C}_{m}^{\text{1}}+{\;C}_{m}^{\text{2}n}=d_{m}\cdot f_{m}+\text{D}\cdot {\hat{A}}_{m}\cdot F_{m}+D\cdot (L-\text{1})\cdot {\hat{A}}_{m}\cdot F_{m}$$ (5)

In the formula, $d_{m}$ is the direct emission factor of the medical institution sector, $F_{m}$ represents the column vector composed of all elements of the final demand $f_{m}$ of the medical institution sector. $A_{m}$ is the column vector of the input of each sector to the medical institution sector (a column in matrix A). For the largest procurement category of medical institution’s purchase of drugs, this study further decomposes the indirect emissions of CO_2_e, that is, the secondary and subsequent indirect emissions of the medical institution. The relationship for the decomposition ${\;C}_{mp}^{\text{2}n}$ of the emissions contained in the purchased drugs is:


$${\;C}_{mp}^{\text{2}n}=D\cdot (L-\text{1})\cdot {\hat{A}}_{P}\cdot {\hat{a}}_{pm}\cdot f_{m}$$ (6)

In the formula, $A_{P}$ represents the input of each sector into the pharmaceutical sector (a column in matrix A). ${\hat{a}}_{pm}$ denotes a diagonal matrix containing all the principal diagonal elements of $a_{mp}$, and $a_{pm}$ indicates the input of the pharmaceutical sector into the production of the medical institution sector per unit of output (an element in matrix A).

### Scope of assessment

**Fig 1:**
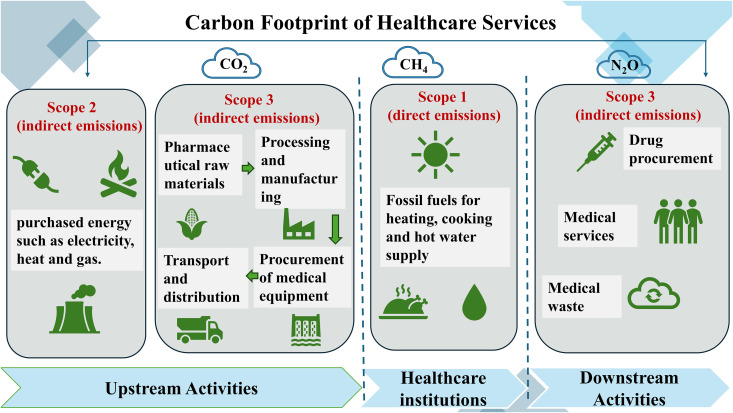
Assessment scope of carbon footprint of healthcare services. Accounting for scope 1, scope 2, and scope 3 emissions, respectively.

According to the Greenhouse Gas Protocol (2022), the carbon footprint in this study is classified into three scopes (Fig 1) [29]. Scope 1 refers to direct GHG emissions generated by hospitals or healthcare organizations, including stationary combustion (e.g., fuel for heating), mobile combustion (e.g., ambulance fleets), process emissions (e.g., chemical reactions in medical production), and fugitive emissions (e.g., refrigerant leaks). Scope 2 encompasses indirect emissions from purchased electricity, steam, heating, or cooling, which occur during energy production but are indirectly driven by the healthcare facility’s energy consumption. Scope 3 includes all other indirect emissions across the healthcare value chain, spanning upstream (e.g., raw material procurement, transportation) and downstream activities (e.g., product use, waste disposal). These emissions, though not directly controlled by healthcare institutions, are intrinsically linked to medical operations.

## Results

### National health expenditure and the carbon footprint of medical services

Table 2 illustrates the changes in national health expenditure by different expenditure categories in 2012 and 2018. The total national health expenditure increased from 2,572 billion CNY in 2012 to 5,174 billion CNY in 2018, representing a growth rate of nearly 101%. Among them, public hospitals accounted for the largest proportion, increasing from 1,355 billion CNY (accounting for 53% of the total) in 2012 to 2,731 billion CNY (54% of the total) in 2018. Private hospitals accounted for the fastest growth, increasing from 99 billion CNY (accounting for 3.2% of the total) in 2012 to 373 billion CNY (12.5% of the total) in 2018, with a growth rate of 276%. This was followed by NHP pharmaceuticals, which experienced a relative decline, falling from 20% (526 billion CNY) of total health expenditure in 2012 to 16% (837 billion CNY) in 2018, despite an increase in absolute expenditure. The health expenditures accounted for 5% to 6% of the Gross Domestic Product (GDP) across different years in China.

**Table 2:** Carbon footprint of Chinese Healthcare Service by expenditure categories in 2012 and 2018.

Expenditure categories		2012						2018					
		Expenditure (billion CNY)	Share of total health expenditure (%)	Direct emission factor (Tons/million CNY)	Total emission factor (Tons/million CNY)	Total GHG emissions (MtCO_2_e)	Share of total GHG emissions (%)	Expenditure (billion CNY)	Share of total health expenditure (%)	Direct emission factor (Tons/million CNY)	Total emission factor (Tons/million CNY)	Total GHG emissions (MtCO_2_e)	Share of total GHG emissions (%)
Healthcare institutions	Public hospitals	1,355	53%	6	112	152	44%	2,731	53%	2	66	181	46%
	Private hospitals	99	4%	6	112	11	3%	373	7%	2	66	25	6%
	Community healthcare	297	12%	6	112	33	10%	586	11%	2	66	39	10%
	Public health	128	5%	6	112	14	4%	266	5%	2	66	18	5%
	Other healthcare institutions	20	1%	6	112	2	1%	45	1%	2	66	3	1%
Pharmaceuticals	NHP pharmaceuticals	526	20%	9	176	93	27%	837	16%	2	99	83	21%
Construction	Construction	103	4%	3	307	33	10%	211	4%	2	184	39	10%
Other services	Management	32	1%	14	93	3	1%	101	2%	6	53	5	1%
	Research	12	0	14	93	1	0.30%	26	1%	6	53	1	0.30%
**Total**		**2,572**	**100%**	**–**	**–**	**342**	**100%**	**5,174**	**100%**	**–**	**–**	**393**	**100%**

Abbreviations: NHP, nonhospital purchase; GHG, greenhouse gas; CNY, Chinese Yuan; Mt, million tonnes; CO_2_e, carbon dioxide equivalent.

Table 2 shows the changes in GHG emissions of the healthcare service by expenditure categories and the share of the healthcare service in total national GHG emissions in 2012 and 2018. The total national GHG emissions of healthcare service increased from 342 MtCO_2_e in 2012 to 393 MtCO_2_e in 2018, with an increase of 15% over the 6-year period in China. Among the various sectors in healthcare services, public hospitals contributed the most significant category of carbon footprint, with their emissions rising from 152 MtCO_2_e (44% of total emissions) in 2012 to 181 MtCO_2_e (46% of total emissions) in 2018. However, private hospitals exhibited the fastest growth trajectory in GHG emissions, with their emissions increasing from 11 MtCO_2_e in 2012 to 25 MtCO_2_e in 2018, representing a 127% increase. Conversely, emissions from NHP pharmaceuticals decreased from 93 MtCO_2_e (27% of total emissions) in 2012 to 83 MtCO_2_e (21% of total emissions) in 2018. During the study period, the GHG emissions of healthcare service accounted for 3.2%−3.7% of the total domestic GHG emissions while healthcare services accounted for 5%–6% of total GDP in China.

### Emission sources of carbon footprint of healthcare services in China

S1 and S2 Figs depict the contributions of 45 economic sectors to the carbon footprint of Chinese healthcare services in 2012 and 2018. By expenditure categories, medical institutions were the largest contributor, with the carbon footprint increasing from 213 MtCO_2_e (62% of the total) in 2012 to –265 MtCO_2_e (67% of the total) in 2018. Regarding emission sources, the carbon footprint of medical institutions was primarily contributed by the facilities that producing and supplying electric power, steam, and hot water, accounting for 40% and 46% of the total carbon emissions is 22,012 in 2018, respectively. Compared to 2012, the carbon emissions from most of these sectors increased in 2018, primarily including the production and supply of electric power, steam and hot water, as well as medical equipment transportation, storage, post office, and telecommunication services. However, there were also a few sectors that experienced a decrease in carbon emissions in 2018, including medical and pharmaceutical products usages, papermaking and paper products, as well as food processing.

### Breakdown of medical institution sector

S3 and S4 Figs illustrate the breakdown of the carbon footprint of the medical institution sector in 2012 and 2018. The first layer represents the decomposition of the carbon footprint within the medical institution sector, where the main emission category is procurement. It increased from 184 MtCO_2_e (86%) in 2012 to 230 MtCO_2_e (87%) in 2018, representing an increase of 25%. The second layer, among the procurements, main emission category is pharmaceuticals, from 120 MtCO_2_e (60%) in 2012 to 140 MtCO_2_e (54%) in 2018, representing a 17% increase in emissions. When exploring further to the third layer, it is found that another important emission category is upstream supply chain of pharmaceuticals, with the emissions increased from 24 MtCO_2_e (30%) in 2012 to 30 MtCO_2_e (31%) in 2018, representing a 25% increase over the period.

### Carbon footprint of public hospitals in China by department

**Fig 2:**
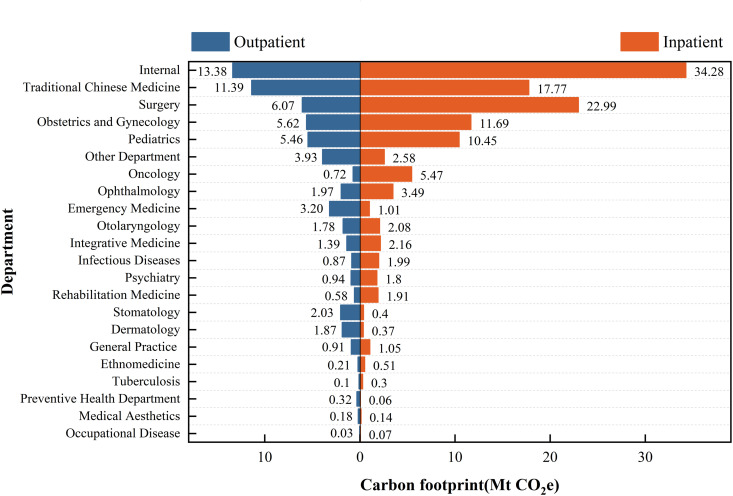
Carbon footprint of public hospitals in China, 2018 by outpatient and inpatient departments. Ranking based on total emissions. Abbreviations: Mt, million tonnes; CO_2_e, carbon dioxide equivalent.

Fig 2 shows the carbon footprint of public hospitals in 2018, breakdown by 22 departments, including both outpatient and inpatient. These 22 departments generated a total carbon footprint of 185.91 MtCO_2_e in 2018. Among them, outpatient departments emitted 63.33 MtCO_2_e, accounting for 34% of the total emissions, while inpatient emitted 122.58 MtCO_2_e, accounting for 66% of the total emissions. The main emission contributors for both outpatient and inpatient departments included the Internal Medicine, the Traditional Chinese Medicine, the Obstetrics and Gynecology, and Pediatrics. For the outpatient departments, the primary emitting departments were the Internal Medicine (13.38 MtCO_2_e [21%]) and the Traditional Chinese Medicine (11.39 MtCO_2_e [18%]). For inpatient departments, the Internal Medicine again emerged as a significant contributor (34.28 MtCO_2_e [28%]), followed by the Surgery (22.99 MtCO_2_e [19%]).

### Carbon footprint of public hospitals in China by hospital level

The carbon footprints of public hospitals at different levels in China, 2018 were also calculated (S1 Table). It was shown that the tertiary hospitals contributed the most (126.50 MtCO_2_e), accounting for approximately 70% of the total emissions among the healthcare services in China. It was followed by the secondary hospitals (28%) and the primary hospitals (2%).

### Carbon footprint of public hospitals in China by specific diseases

**Fig 3:**
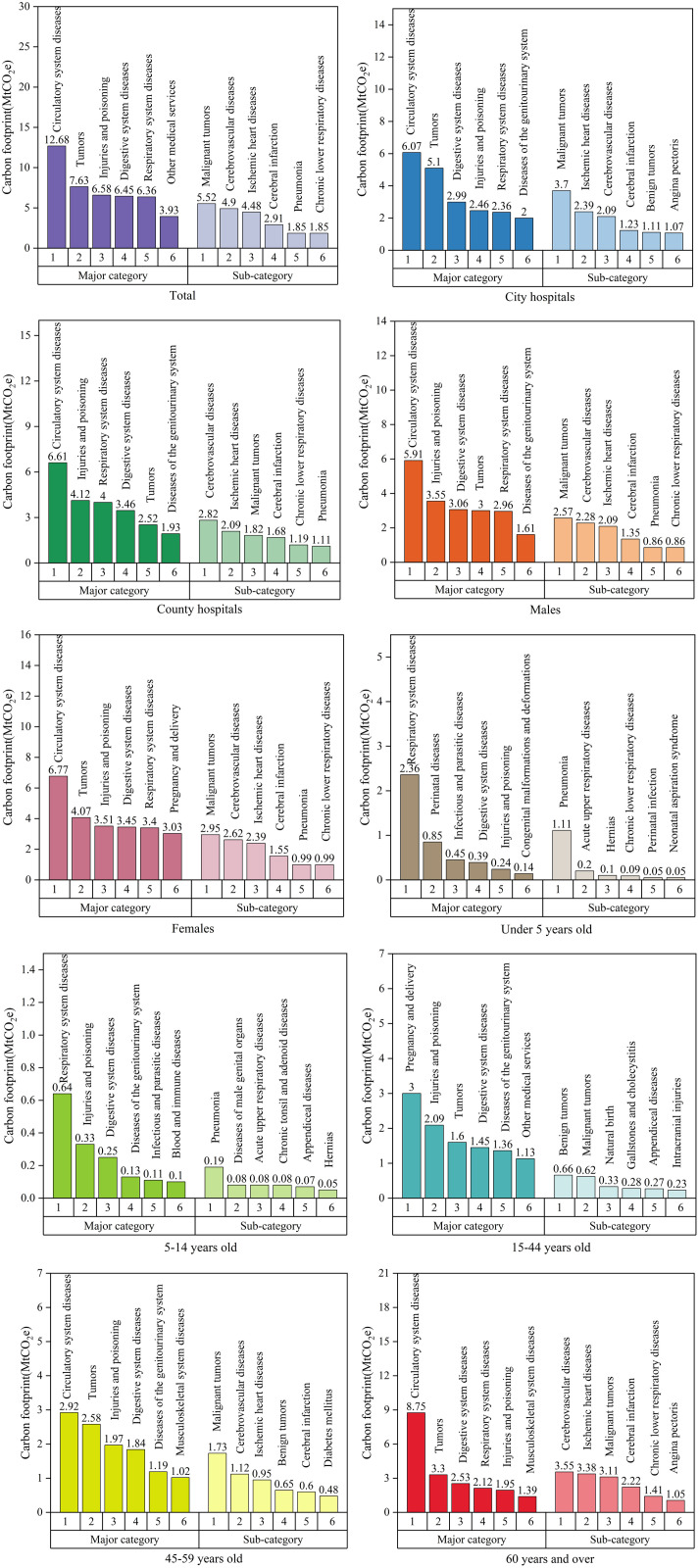
Carbon footprints of public hospitals attributed to specific diseases in 2018. Stratified by city/county, gender, and age further analyzed and ranked for each type of stratification. The figure highlights in particular the top six diseases with the largest carbon footprint contributions and shows the carbon footprint contributions of these diseases at both the major category and subcategory levels. Abbreviations: Mt, million tonnes; CO_2_e, carbon dioxide equivalent.

We analyzed the carbon footprints of public hospitals attributed to specific diseases in 2018 (Fig 3 and S2 Table), including 20 major categories and 154 subcategories of diseases, and stratified the data by city and county, gender, and age. The total carbon footprint generated by various diseases was 65.15 MtCO_2_e in 2018. In terms of the overall contribution of the carbon footprint by disease category, the major contributors were circulatory system diseases (12.68 MtCO_2_e [19%]), followed by tumors (7.63 MtCO_2_e [12%]), injuries and poisonings (6.58 MtCO_2_e [10%]). Further subdivided into subcategories of diseases, the main contributors were malignant tumors (5.52 MtCO_2_e [8%]), cerebrovascular diseases (4.90 MtCO_2_e [8%]), and ischemic heart diseases (4.48 MtCO_2_e [7%]).

Stratified analysis indicated that urban hospitals and county-level hospitals produced a carbon footprint of 31.86 MtCO_2_e and 33.30 MtCO_2_e in 2018, respectively (Fig 3 and S2 Table). For urban hospitals, the primary contributors, by disease category, were circulatory system diseases (6.07 MtCO_2_e [10%]), tumors (5.10 MtCO_2_e [8%]), and digestive system diseases (2.99 MtCO_2_e [5%]). For county-level hospitals, the main contributors included circulatory system diseases (6.61 MtCO_2_e [10%]), injuries and poisoning (4.12 MtCO_2_e [6%]), as well as respiratory system diseases (4.00 MtCO_2_e [6%]). Overall, across both urban and county-level hospitals, malignant tumors, ischemic heart diseases, and cerebrovascular diseases also emerged as key contributors to the carbon footprint.

The carbon footprint generated by male and female hospitalizations was 30.35 MtCO_2_e and 34.80 MtCO_2_e, respectively (Fig 3 and S2 Table). For male patients, the main contributors, by disease category, were circulatory system diseases (5.91 MtCO_2_e [9%]), injuries and poisoning (3.06 MtCO_2_e [5%]), and digestive system diseases (3.00 MtCO_2_e [5%]). For female patients, the main contributors were circulatory system diseases (6.77 MtCO_2_e [10%]), tumors (3.72 MtCO_2_e [7%]), and injuries and poisoning (3.51 MtCO_2_e [5%]). For both males and females, malignant tumors, cerebrovascular diseases, and ischemic heart diseases also emerged as key contributors to the carbon footprint.

Age-specific carbon footprint shows that the patients aged 5 years and under generated a carbon footprint of 6.65 MtCO_2_e (10%) in 2018 (Fig 3 and S2 Table), with respiratory system diseases being the primary contributor (2.36 MtCO_2_e [4%]), and pneumonia being the main subcategory contributor for this age group (1.11 MtCO_2_e [2%]). Patients aged 5–14 years produced 2.48 MtCO_2_e (4%) emissions, with respiratory system diseases also being the main contributor (0.64 MtCO_2_e [1%]), and pneumonia being the primary subcategory contributor (0.19 MtCO_2_e [0.3%]). Patients aged 15–44 years contributed 15.77 MtCO_2_e (24%) emissions, with pregnancy and childbirth diseases being the main contributor (3.00 MtCO_2_e [5%]), and benign tumors (0.66 MtCO_2_e [1%]) being the main subcategory contributor. Patients aged 45–59 years produced emissions of 14.98 MtCO_2_e (23%), with circulatory system diseases being the main contributor (2.33 MtCO_2_e [4%]), and malignant tumors being the primary subcategory contributor (1.38 MtCO_2_e [2%]). And patients aged 60 years and above generated 25.28 MtCO_2_e (39%), with the main contributor being circulatory system diseases (8.75 MtCO_2_e [13%]), and ischemic heart diseases (3.38 MtCO_2_e [5%]) as the main subcategory contributor.

## Discussion

Overall, the total carbon footprint of Chinese healthcare service showed an upward trend during the years 2012 and 2018. While healthcare spending was 2,572 billion CNY in 2012 and 5,174 billion CNY in 2018, accounted for 5% and 6% of GDP, respectively, our study of carbon footprint analyses of the Chinese healthcare service, using EEIOA assessment method, showed that it was 342 MtCO_2_e in 2012 and 393 MtCO_2_e in 2018, accounting for 3.2% and 3.7% of the total domestic GHG emissions, respectively. This result indicated that healthcare services accounted for a relatively small component of total GHG emissions, but its carbon intensity per unit of GDP was relatively high considering its share in the national economy in China. This may reflect that there is room for improvement in energy use efficiency and carbon emission control in the Chinese healthcare services sector. This study found that with the steady growth in demands for healthcare services, both health expenditures and GHG emissions were on the rise in China. This may be attributed to increased expenditure on medical facilities and equipment, with most power supply for healthcare services are still from fossil fuels in China. However, such relationships may vary in different countries. For example, the Austrian healthcare service had reduced its carbon footprint by 14% between 2005 and 2014 [20], primarily due to the increasing usages of renewable energy. This first national comprehensive finding not only quantified the carbon emissions of the Chinese healthcare service but also provided an important scientific evidence and reference for the formulation of future targeted policies for mitigating carbon emissions within the healthcare sector.

Research has indicated that the carbon footprint of pharmaceuticals (prescribed medications) purchased by medical institutions was continuously increasing, while the carbon footprint of nonprescribed medication was showing a decreasing trend. This may be due to the amendment in healthcare policies. The government has controlled the price of medications and is adjusting the reimbursement rates of medical insurance [30]. Additionally, the government has restricted the sales of nonprescription medications. Overall, pharmaceuticals accounted for approximately 57% of the carbon footprint of the entire Chinese healthcare service in 2018, including prescription medications (140 MtCO_2_e [36%]) and over-the-counter drugs (83 MtCO_2_e [21%]). To reduce the carbon footprint, we need to reduce the number of drug prescriptions [31], minimize the production of unused drugs [32], and encourage clinicians to choose low-carbon drug alternatives [22]. These are all effective ways to reduce the carbon footprint from medication utilizations in healthcare services in China.

The findings showed that between 2012 and 2018, the emissions generated in the supply chain resulting from the purchase of goods and services by the medical institutions were continuously increasing, accounting for 54% and 59% of the national carbon footprint, respectively. Therefore, it is crucial to reduce emissions in the medical supply chain. Methods to reduce supply chain emissions include both upstream (at the supplier end) and downstream (at the production and consumption ends) strategies [21]. Upstream, adopt green procurement by prioritizing low-carbon suppliers, promoting renewable energy, and eco-friendly materials [33]; enhance energy efficiency with energy-saving medical equipment and technology [34]. Downstream, optimize production processes, such as reducing medical waste and remanufacturing medical devices [35]; improve storage management with energy-efficient lighting and air conditioning systems to decrease energy consumption in warehouses [36]; strengthen circular economy by encouraging recycling and reuse of medical equipment and clothing, reducing the use of disposable products [2]; promote green medical buildings by using low-carbon construction materials to cut demand for high-carbon emitting building materials [37]. The pharmaceutical industry should fully leverage its value chain to promote collaborative carbon reduction efforts both upstream and downstream.

Our results demonstrated that the total carbon footprint of all departments in public hospitals in 2018 was 185.91 MtCO_2_e, with inpatient services emitting approximately twice as much as outpatient services. This may be attributed to the severe conditions of inpatients, massive workloads involving extensive use of medical equipment, medications, and energy. The Internal Medicine Department had the highest emissions, possibly due to the long-term medication usage and complex diagnostic procedures required for various diseases. Traditional Chinese Medicine Department ranked second, likely due to the widespread use of herbal medicines, generating emissions throughout the long process from herb collection, processing, and application for treatment. To reduce carbon emissions, hospitals should prioritize the selection of low-emission medical equipment, such as anesthetic gases [33,34] and inhalers [35,38]. Furthermore, (0.66 tCO_2_e) per capita emissions for inpatients were ~33 times higher than those for outpatients (0.02 tCO_2_e). Therefore, preventing the unnecessary conversion of outpatients to inpatients can significantly reduce the carbon footprint of the healthcare industry [21].

Our research indicated that tertiary hospitals were the primary emission contributor among the healthcare services in China. To reduce carbon emissions, two primary strategies might be implemented. Firstly, it is necessary to redesign a tiered medical system, including to establish a two-way referral system and to define the responsibilities of medical institutions at all levels which encourage county-level hospitals to play an essential role in healthcare services [39]. Secondly, a better healthcare service system to coordinate health services at different levels, such as a telemedicine service system to reduce patients’ transportation from township and county hospitals to tertiary hospitals [40]. It should be acknowledged that the indirect carbon footprint for the patients’ and their family members cross-region transportation and accommodations are not calculated in this study which needs to be explored in future.

The carbon emissions from disease management in 2018 were about 65.15 MtCO_2_e, accounting for 36% of the carbon footprint of public hospitals in China. Our study shows that emissions are slightly higher for patients in county hospitals than in urban hospitals. However, the carbon emissions from tumors in urban hospitals were approximately twice those in county-level hospitals. This may be due to urban hospitals typically having more specialized tumor diagnostic and treatment techniques. We can enhance local medical services and optimize medical resource allocation. This study used the proportional allocation method based on gender hospitalization rate for stratified analysis. The study showed that female patient’s emissions are slightly higher than those of males. As women are at higher risk for certain diseases, such as oncology-related diseases (breast, cervical cancer, etc.), as well as pregnancy and childbirth-related diseases (high blood pressure in pregnancy, post-partum hemorrhage, etc.) [41], gender differences need to be considered in the formulation of public health strategies to provide more targeted disease screening and prevention services for women.

In terms of age distribution, patients aged 60 years and above have the highest level of carbon footprint compared to other age groups. This is because older people have many comorbidities, such as cardiovascular diseases and diabetes, which require prolonged treatment and monitoring. Therefore, providing comprehensive healthcare services to the older is critical to reducing the carbon footprint of healthcare services. The list goes on, for example, to enhance early screening and intervention for chronic diseases, and optimize medication management to reduce unnecessary hospitalization and treatment. However, as China’s population ages, older patients are increasingly contributing to the overall carbon footprint, suggesting that future carbon assessments must take into account the increased demand for healthcare services resulting from a growing older population, enhanced management of chronic diseases, and changes in lifestyle, all of which may lead to increased energy consumption and carbon emissions.

According to the World Health Organization’s Alliance for Transformative Action on Climate and Health (ATACH), China has not yet made a public commitment to develop climate-resilient and low-carbon sustainable healthcare systems, but is actively taking measures to address carbon emissions within the healthcare sector. Firstly, the national “dual carbon” strategy extends to include public institutions, including hospitals, in the energy-saving and emission-reduction efforts outlined in the “Energy Conservation and Carbon Reduction Action Plan for 2024–2025” [42]. This plan calls for a 50% photovoltaic rooftop coverage rate for new hospitals. Secondly, green hospital construction standards are being implemented, with regions like Guangdong and Shanghai requiring top-tier hospitals to adopt intelligent energy management systems and phase out coal-fired boilers [43]. Thirdly, there are attempts to decarbonize the supply chain, with China participating in the “Sustainable Market Initiative” Health Systems Working Group and collaborating with multinational pharmaceutical companies to promote green electricity procurement, exemplified by a 700-megawatt renewable energy project in Jiangsu and Guangdong [44]. Moving forward, decarbonization should be prioritized in healthcare policies. Firstly, we need to develop more precise methods for estimating carbon emissions and explore the differences in carbon emissions among various regions and types of medical services [15]. Secondly, a carbon peak roadmap for the healthcare industry should be established, setting clear targets for carbon emission peaks in medical institutions before 2030, and detailing reduction pathways in areas such as construction, equipment, and supply chains [45]. Thirdly, strengthen the data foundation and assessment mechanisms, establish healthcare carbon accounting standards, and incorporate carbon emission indicators into hospital rating systems (for instance, by drawing on the carbon disclosure requirements for suppliers in the UK’s NHS) [45]. In summary, a growing number of countries and regions have formally committed to developing climate-resilient and low-carbon, sustainable health systems, and Chinese policymakers should also consider incorporating decarbonization into health policy priorities.

This study provides a comprehensive estimation of the carbon footprint among healthcare services in China and the carbon footprint trends in 2012 and 2018, using public available data. Its strength of this study include: firstly, it identified primary carbon footprint contributor in the China healthcare services which laid solid scientific evidence for policymakers for their mitigation strategy development; secondly, we also examined the variations of carbon footprint contributions within hospital departments and among different disease categories which also provide evidence for their mitigation practice development.

However, the limitations of this study should also be acknowledged. Firstly, the national IOT is updated every 5 years, and CH_4_ and N_2_O data have not been updated, hence calculations can only be made for 2012 and 2018. Secondly, a competitive IOT was used, which may overestimate the impact of final demand on domestic carbon emissions. The drawback is that the competitive table does not distinguish between imports in intermediate and final output, with the production of these imports occurring abroad and their associated carbon emissions also occurring overseas, especially for medical equipment and imported pharmaceuticals, thus using such tables can easily overestimate the impact of final demand on domestic emissions. Thirdly, the carbon emission intensity of medical institutions may also underestimate the carbon footprint of hospitals and overestimate that of other categories. This is because there are significant differences in carbon emission intensity among different types of medical institutions. Fourthly, the agricultural sector’s GHGs mainly come from methane emissions from animals and rice, but the main raw materials for the pharmaceutical sector come from corn, thus also potentially overestimating emissions from the agricultural sector. Fifthly, carbon emissions from anesthetic gases and metered-dose inhalers were not included in the assessment due to data limitations, and therefore, the carbon emissions from healthcare services may have been underestimated. Sixthly, sensitivity analyses are not possible for the time being as the post-2017 IOTs no longer contain calculation error terms. Therefore, future research could consider using other methods or data sources to address this limitation.

We assessed emission hotspots and structural pathways in different time periods and conducted in-depth analyses of public hospitals. These findings enable us to propose specific measures for the pharmaceutical industry, supply chain, internal medicine, tertiary hospitals and specific medical disease types. It can quantify the data of the healthcare service and analyze the carbon emission data of each link, identify potential emission reduction links and methods, better understand the impact of this industry on global climate change, and provide reference for other industries.

## Supporting information

S1 Table Carbon footprints of tiered public hospitals in 2018. (PDF)

S2 Table The carbon footprint of public hospitals attributed to specific diseases in 2018. (PDF)

S1 Fig The contribution of 45 economic sectors to the carbon footprint of Chinese Healthcare Service in 2012. (TIF)

S2 Fig The contribution of 45 economic sectors to the carbon footprint of Chinese Healthcare Service in 2018. (TIF)

S3 Fig Breakdown of the carbon footprint of the healthcare institutions sector in 2012. (TIF)

S4 Fig Breakdown of the carbon footprint of the healthcare institutions sector in 2018. (TIF)

S1 CHEERS checklist. (PDF)

## Abbreviations

ATACH: alliance for transformative action on climate and health
EEIOA: environmentally extended input–output analysis
GDP: gross domestic product
GHG: global greenhouse gas
GWP: global warming potential
IOTs: input–output tables
NBS: National Bureau of Statistics
NHP: non-hospital purchased
SPA: structural path analysis
