# Supplementary material for: Carbon footprint of the Chinese healthcare service: An environmentally extended input–output analysis
Source: PLoS Med. 2025 Sep 24;22(9):e1004738. doi: 10.1371/journal.pmed.1004738 (PMC12459823; doi:10.1371/journal.pmed.1004738)
Supplement: S1 Table — (PDF) [file pmed.1004738.s001.pdf]

S1 Table. Carbon footprints of tiered public hospitals in 2018. Abbreviations: CNY, Chinese Yuan. Mt, million tonnes. CO_2e, carbon dioxide equivalent.

**Hospital level**	**Expenditure (billion CNY)**	**Carbon footprint ($MtCO_2e$)**
Total public hospitals	2731	180.55
Tertiary hospitals	1914	126.50
Secondary hospitals	759	50.17
Primary hospitals	32	2.09
