# Supplementary material for: Carbon footprint of the Chinese healthcare service: An environmentally extended input–output analysis
Source: PLoS Med. 2025 Sep 24;22(9):e1004738. doi: 10.1371/journal.pmed.1004738 (PMC12459823; doi:10.1371/journal.pmed.1004738)
Supplement: S2 Table — (PDF) [file pmed.1004738.s002.pdf]

S2 Table. The carbon footprint of public hospitals attributed to specific diseases in 2018. Major categories: diseases in bold font. Sub-categories: diseases with un-bolded font. Unit: $MtCO_2e$. Abbreviations: Mt, million tonnes. CO_2e, carbon dioxide equivalent.

ID	Diseases	ICD-10 code	Total carbon footprint	City hospitals	County hospitals	Males	Females	Under 5 years old	5-14 years old	15-44 years old	45-59 years old	60 years and over
000	**Total**	A00-T98，Z00-Z99	65.15	31.86	33.30	30.35	34.80	6.65	2.48	15.77	14.98	25.28
001	**1. Infectious and parasitic diseases**	A00-B99	1.29	0.55	0.74	0.60	0.69	0.45	0.11	0.24	0.21	0.29
002	Enteric infectious diseases	A00-A09	0.07	0.02	0.05	0.03	0.04	0.04	0.00	0.01	0.01	0.01
003	Cholera	A00	0.00	0.00	0.00	0.00	0.00	0.00	0.00	0.00	0.00	0.00
004	Typhoid and paratyphoid fever	A01	0.00	0.00	0.00	0.00	0.00	0.00	0.00	0.00	0.00	0.00
005	Bacterial dysentery	A03	0.00	0.00	0.00	0.00	0.00	0.00	0.00	0.00	0.00	0.00
006	Tuberculosis	A15-A19	0.37	0.18	0.19	0.17	0.20	0.00	0.00	0.12	0.09	0.15
007	Tuberculosis	A15-A16	0.25	0.11	0.14	0.12	0.14	0.00	0.00	0.08	0.07	0.11
008	Diphtheria	A36	0.00	0.00	0.00	0.00	0.00	0.00	0.00	0.00	0.00	0.00
009	Pertussis	A37	0.00	0.00	0.00	0.00	0.00	0.00	0.00	0.00	0.00	0.00
010	Scarlet fever	A38	0.00	0.00	0.00	0.00	0.00	0.00	0.00	0.00	0.00	0.00
011	Sexually transmitted diseases	A50-A64	0.01	0.00	0.00	0.00	0.00	0.00	0.00	0.00	0.00	0.00
012	Syphilis	A50-A53	0.00	0.00	0.00	0.00	0.00	0.00	0.00	0.00	0.00	0.00
013	Gonococcal infections	A54	0.00	0.00	0.00	0.00	0.00	0.00	0.00	0.00	0.00	0.00
014	Encephalitis B	A83.0	0.00	0.00	0.00	0.00	0.00	0.00	0.00	0.00	0.00	0.00
015	Typhus	A75	0.01	0.00	0.01	0.00	0.01	0.00	0.00	0.00	0.00	0.00
016	Viral hepatitis	B15-B19	0.14	0.07	0.07	0.07	0.07	0.00	0.00	0.07	0.05	0.02
017	HIV	B20-B24	0.03	0.01	0.02	0.02	0.02	0.00	0.00	0.01	0.01	0.01

018	Schistosomiasis	B65	0.00	0.00	0.00	0.00	0.00	0.00	0.00	0.00	0.00	0.00
019	Filariasis	B74	0.00	0.00	0.00	0.00	0.00	0.00	0.00	0.00	0.00	0.00
020	Hookworm diseases	B76	0.00	0.00	0.00	0.00	0.00	0.00	0.00	0.00	0.00	0.00
021	**2. Tumors**	C00-D48	7.63	5.10	2.52	2.38	3.72	0.08	0.07	1.60	2.58	3.30
022	Malignant tumor meter	C00-C97	5.52	3.70	1.82	1.96	2.45	0.03	0.03	0.62	1.73	3.11
023	Nasopharyngeal malignant tumor	C11	0.05	0.03	0.02	0.02	0.03	0.00	0.00	0.01	0.02	0.02
024	Esophageal malignant tumors	C15	0.25	0.13	0.12	0.12	0.14	0.00	0.00	0.00	0.05	0.20
025	Malignant tumor of stomach	C16	0.50	0.29	0.22	0.24	0.27	0.00	0.00	0.02	0.12	0.36
026	Malignant tumor of small intestine	C17	0.03	0.02	0.01	0.01	0.01	0.00	0.00	0.00	0.01	0.02
027	Malignant tumors of the colon	C18	0.39	0.25	0.13	0.18	0.21	0.00	0.00	0.03	0.10	0.25
028	Malignant tumors of rectosigmoid junction, rectum, anus and anal canal	C19-C21	0.39	0.24	0.14	0.18	0.21	0.00	0.00	0.02	0.11	0.25
029	Malignant tumor of liver and intrahepatic bile ducts	C22	0.40	0.26	0.14	0.19	0.22	0.00	0.00	0.04	0.15	0.21
030	Malignant tumor of larynx	C32	0.04	0.03	0.01	0.02	0.02	0.00	0.00	0.00	0.01	0.03
031	Malignant tumors of trachea, bronchus, lungs	C33-C34	0.99	0.63	0.36	0.46	0.53	0.00	0.00	0.04	0.26	0.69
032	Malignant tumors of bone and articular cartilage	C40-C41	0.02	0.01	0.01	0.01	0.01	0.00	0.00	0.00	0.00	0.01
033	Malignant tumor of breast	C50	0.37	0.26	0.11	0.17	0.20	0.00	0.00	0.08	0.18	0.10
034	Malignant tumor of female genital organs	C51-C58	0.36	0.25	0.11	0.00	0.29	0.00	0.00	0.06	0.18	0.12
035	Malignant tumor of male genital organs	C60-C63	0.12	0.09	0.04	0.10	0.00	0.00	0.00	0.00	0.01	0.11

036	Malignant tumor of urinary tract	C64-C68	0.25	0.18	0.07	0.12	0.13	0.00	0.00	0.02	0.06	0.17
037	Malignant tumor of brain	C71	0.07	0.06	0.02	0.03	0.04	0.00	0.00	0.02	0.02	0.02
038	Leukemia	C91-C95	0.19	0.14	0.05	0.09	0.10	0.01	0.01	0.05	0.05	0.07
039	Carcinoma in situ	D00-D09	0.13	0.10	0.04	0.06	0.07	0.00	0.00	0.04	0.05	0.03
040	Carcinoma in situ of the uterine cervix	D09	0.06	0.05	0.01	0.00	0.05	0.00	0.00	0.03	0.03	0.01
041	Benign tumors	D10-D36	1.63	1.11	0.52	0.35	0.95	0.03	0.03	0.66	0.65	0.27
042	Benign skin tumors	D22-D23	0.02	0.01	0.01	0.01	0.01	0.00	0.00	0.01	0.00	0.00
043	Benign breast tumors	D24	0.17	0.13	0.04	0.00	0.14	0.00	0.00	0.12	0.04	0.01
044	Uterine smooth muscle tumor	D25	0.43	0.26	0.17	0.00	0.34	0.00	0.00	0.17	0.25	0.01
045	Benign ovarian tumors	D27	0.12	0.08	0.03	0.00	0.09	0.00	0.00	0.07	0.03	0.01
046	Benign prostate tumors	D29.1	0.00	0.00	0.00	0.00	0.00	0.00	0.00	0.00	0.00	0.00
047	Benign thyroid tumor	D34	0.06	0.03	0.02	0.03	0.03	0.00	0.00	0.02	0.02	0.01
048	Junctional malignant and dynamically unknown tumors	D37-D48	0.34	0.20	0.14	0.16	0.18	0.00	0.00	0.07	0.10	0.17
049	**3.Blood, hematopoietic organ and immune diseases**	D50-D89	0.45	0.26	0.19	0.21	0.24	0.04	0.10	0.09	0.08	0.14
050	Anemia	D50-D64	0.25	0.13	0.12	0.11	0.13	0.02	0.04	0.05	0.05	0.09
051	**4. Endocrine, nutritional and metabolic diseases**	E00-E90	1.85	1.03	0.82	0.86	0.99	0.03	0.04	0.30	0.65	0.83
052	Hyperthyroidism	E05	0.05	0.04	0.02	0.03	0.03	0.00	0.00	0.02	0.02	0.01
053	Diabetes mellitus	E10-E14	1.33	0.71	0.62	0.62	0.71	0.01	0.01	0.17	0.48	0.66
054	**5. Mental and behavioral disorders**	F00-F99	0.35	0.19	0.16	0.16	0.18	0.01	0.01	0.12	0.11	0.09

055	Dependent substance-induced mental and behavioral disorders	F11 -F19	0.01	0.00	0.01	0.01	0.01	0.00	0.00	0.01	0.00	0.00
056	Alcohol-induced mental and behavioral disorders	F10	0.01	0.00	0.01	0.01	0.01	0.00	0.00	0.01	0.00	0.00
057	Schizophrenia, schizotypal and delusional disorders	F20-F29	0.08	0.04	0.04	0.04	0.04	0.00	0.00	0.04	0.03	0.01
058	Affective disorders	F30-F39	0.05	0.04	0.01	0.02	0.03	0.00	0.00	0.02	0.01	0.01
059	**6. Nervous system diseases**	G00-G99	1.80	0.92	0.88	0.84	0.96	0.08	0.06	0.23	0.48	0.94
060	Inflammatory diseases of the central nervous system	G00-G09	0.09	0.06	0.04	0.04	0.05	0.02	0.03	0.02	0.01	0.01
061	Parkinson's disease	G20	0.06	0.04	0.02	0.03	0.03	0.00	0.00	0.00	0.01	0.05
062	Epilepsy	G40-G41	0.12	0.07	0.05	0.06	0.06	0.01	0.02	0.03	0.02	0.03
063	**7. Eye and appendage diseases**	H00-H59	1.06	0.62	0.45	0.49	0.57	0.02	0.03	0.10	0.22	0.70
064	Lens disorders	H25-H28	0.59	0.32	0.27	0.27	0.31	0.00	0.00	0.01	0.08	0.49
065	Age-related cataracts	H25	0.43	0.22	0.21	0.20	0.23	0.00	0.00	0.00	0.04	0.39
066	Retinal detachment and breakage	H33	0.06	0.06	0.00	0.03	0.03	0.00	0.00	0.02	0.02	0.02
067	Glaucoma	H40-H42	0.07	0.04	0.02	0.03	0.04	0.00	0.00	0.01	0.02	0.04
068	**8. Ear and mastoid disorders**	H60-H95	0.42	0.20	0.22	0.20	0.22	0.01	0.02	0.10	0.13	0.16
069	Middle ear and mastoid diseases	H65-H75	0.12	0.07	0.05	0.05	0.06	0.01	0.02	0.04	0.03	0.02
070	**9. Circulatory system diseases**	I00-I99	12.68	6.07	6.61	5.91	6.77	0.10	0.06	0.85	2.92	8.75
071	Acute rheumatic fever	I00-I02	0.00	0.00	0.00	0.00	0.00	0.00	0.00	0.00	0.00	0.00
072	Chronic rheumatic heart diseases	I05-I09	0.08	0.04	0.05	0.04	0.04	0.00	0.00	0.00	0.03	0.05
073	Hypertension	I10-I15	0.67	0.32	0.35	0.31	0.36	0.00	0.00	0.06	0.19	0.42
074	Hypertensive heart and kidney diseases	I11-I13	0.10	0.05	0.05	0.05	0.05	0.00	0.00	0.01	0.02	0.08

075	Ischemic heart diseases	I20-I25	4.48	2.39	2.09	2.09	2.39	0.02	0.00	0.13	0.95	3.38
076	Angina pectoris	I20	1.46	1.07	0.39	0.68	0.78	0.00	0.00	0.04	0.36	1.05
077	Acute myocardial infarction	I21-I22	1.10	0.73	0.37	0.51	0.59	0.00	0.00	0.06	0.28	0.76
078	Pulmonary embolism	I26	0.06	0.05	0.02	0.03	0.03	0.00	0.00	0.00	0.01	0.05
079	Arrhythmia	I47-I49	0.61	0.37	0.24	0.29	0.33	0.00	0.01	0.08	0.16	0.36
080	Heart Failure	I50	0.41	0.17	0.24	0.19	0.22	0.01	0.00	0.01	0.05	0.34
081	Cerebrovascular Diseases	I60-I69	4.90	2.09	2.82	2.28	2.62	0.03	0.00	0.20	1.12	3.55
082	Intracranial hemorrhage	I60-I62	1.13	0.50	0.63	0.53	0.60	0.01	0.01	0.09	0.34	0.68
083	Cerebral Infarction	I63	2.91	1.23	1.68	1.35	1.55	0.01	0.00	0.07	0.60	2.22
084	Cerebral artery occlusion and stenosis	I66	0.07	0.04	0.03	0.03	0.04	0.00	0.00	0.00	0.02	0.05
085	Phlebitis and thrombosis	I80-I82	0.14	0.10	0.04	0.07	0.08	0.00	0.00	0.02	0.04	0.09
086	Varicose veins of the lower extremities	I83	0.16	0.08	0.07	0.07	0.09	0.00	0.00	0.02	0.07	0.07
087	**10. Respiratory system diseases**	J00-J99	6.36	2.36	4.00	2.96	3.40	2.36	0.64	0.55	0.69	2.12
088	Acute upper respiratory tract infections	J00-J06	0.35	0.09	0.25	0.16	0.19	0.20	0.08	0.03	0.02	0.02
089	Influenza	J10-J11	0.01	0.01	0.01	0.01	0.01	0.01	0.00	0.00	0.00	0.00
090	Human avian influenza	J09.x01	0.00	0.00	0.00	0.00	0.00	0.00	0.00	0.00	0.00	0.00
091	Pneumonia	J12-J18	1.85	0.74	1.11	0.86	0.99	1.11	0.19	0.10	0.12	0.33
092	Chronic sinusitis	J32.9	0.15	0.08	0.07	0.07	0.08	0.00	0.02	0.06	0.05	0.03
093	Chronic tonsil and adenoid disease	J35	0.15	0.10	0.05	0.07	0.08	0.03	0.08	0.04	0.01	0.00
094	Chronic lower respiratory tract disease	J40-J47	1.85	0.66	1.19	0.86	0.99	0.09	0.03	0.07	0.24	1.41

095	Asthma	J45-J46	0.12	0.05	0.06	0.05	0.06	0.01	0.01	0.02	0.04	0.04
096	Lung diseases caused by external substances	J60-J70	0.08	0.04	0.04	0.04	0.04	0.00	0.00	0.00	0.02	0.05
097	**11. Digestive system diseases**	K00-K93	6.45	2.99	3.46	3.00	3.45	0.39	0.25	1.45	1.84	2.53
098	Oral Diseases	K00-K14	0.13	0.07	0.06	0.06	0.07	0.02	0.02	0.04	0.03	0.03
099	Gastric and duodenal ulcers	K25-K27	0.33	0.14	0.19	0.15	0.17	0.00	0.00	0.07	0.10	0.15
100	Appendix Diseases	K35-K38	0.61	0.22	0.39	0.28	0.32	0.01	0.07	0.27	0.14	0.11
101	Hernia	K40-K46	0.52	0.23	0.29	0.24	0.28	0.10	0.05	0.06	0.10	0.22
102	Inguinal hernia	K40	0.44	0.19	0.25	0.21	0.24	0.09	0.05	0.05	0.08	0.18
103	Bowel obstruction	K56	0.29	0.13	0.16	0.13	0.15	0.03	0.01	0.04	0.06	0.15
104	Alcoholic liver diseases	K70-K77	0.04	0.02	0.02	0.02	0.02	0.00	0.00	0.01	0.02	0.01
105	Cirrhosis	K74.1	0.38	0.21	0.17	0.18	0.20	0.00	0.00	0.05	0.16	0.17
106	Cholelithiasis and cholecystitis	K80-K81	1.32	0.66	0.67	0.62	0.71	0.01	0.00	0.28	0.43	0.60
107	Acute pancreatitis	K85	0.36	0.17	0.19	0.17	0.19	0.00	0.00	0.13	0.11	0.11
108	**12. Skin and subcutaneous tissue diseases**	L00-L99	0.38	0.21	0.18	0.18	0.20	0.03	0.04	0.12	0.09	0.12
109	Dermatitis and eczema	L20-L30	0.05	0.03	0.02	0.02	0.02	0.00	0.00	0.01	0.01	0.02
110	Psoriasis	L40	0.02	0.02	0.00	0.01	0.01	0.00	0.00	0.01	0.01	0.00
111	Urticaria	L50	0.02	0.01	0.01	0.01	0.01	0.00	0.01	0.01	0.00	0.00
112	**13. Musculoskeletal system diseases**	M00-M99	3.11	1.62	1.49	1.45	1.66	0.04	0.04	0.62	1.02	1.39
113	Inflammatory polyarthritis	M05-M14	0.26	0.15	0.11	0.12	0.14	0.00	0.00	0.04	0.08	0.13
114	Rheumatoid arthritis	M06.9	0.13	0.09	0.04	0.06	0.07	0.00	0.00	0.02	0.05	0.06
115	Gout	M10.901	0.06	0.03	0.03	0.03	0.03	0.00	0.00	0.01	0.02	0.03
116	Other joint diseases	M20-M25	0.45	0.27	0.18	0.21	0.24	0.00	0.00	0.02	0.12	0.30

117	Systemic connective tissue diseases	M30-M36	0.19	0.16	0.03	0.09	0.10	0.01	0.01	0.07	0.06	0.04
118	Systemic lupus erythematosus	M32	0.08	0.06	0.01	0.04	0.04	0.00	0.00	0.04	0.02	0.01
119	Spondylolisthesis	M47	0.28	0.11	0.17	0.13	0.15	0.00	0.00	0.06	0.12	0.11
120	Intervertebral disc diseases	M50-51	0.64	0.26	0.39	0.30	0.34	0.00	0.00	0.13	0.23	0.28
121	Diseases of bone density and bone structure	M80-M85	0.25	0.14	0.10	0.11	0.13	0.00	0.00	0.01	0.03	0.20
122	Osteoporosis	M81.9	0.19	0.11	0.08	0.09	0.10	0.00	0.00	0.00	0.02	0.17
123	Osteomyelitis	M86	0.02	0.01	0.01	0.01	0.01	0.00	0.00	0.01	0.01	0.01
124	**14. Genitourinary diseases**	N00-N99	3.93	2.00	1.93	1.61	1.53	0.07	0.13	1.36	1.19	1.17
125	Glomerular diseases	N00-N08	0.19	0.13	0.05	0.09	0.10	0.00	0.01	0.06	0.06	0.05
126	Pyelonephritis	N16.4	0.04	0.02	0.02	0.02	0.02	0.00	0.00	0.01	0.01	0.01
127	Kidney failure	N17-N19	0.80	0.45	0.35	0.37	0.43	0.00	0.00	0.15	0.27	0.37
128	Urolithiasis	N20-N23	0.46	0.18	0.28	0.22	0.25	0.00	0.00	0.15	0.17	0.14
129	Cystitis	N30	0.03	0.01	0.01	0.01	0.01	0.00	0.00	0.01	0.01	0.01
130	Urethral stenosis	N35	0.01	0.01	0.01	0.01	0.01	0.00	0.00	0.00	0.00	0.01
131	Diseases of the male reproductive organs	N40-N51	0.47	0.22	0.26	0.38	0.00	0.03	0.08	0.08	0.05	0.23
132	Prostate hyperplasia	N40	0.32	0.16	0.16	0.26	0.00	0.00	0.00	0.00	0.02	0.30
133	Breast diseases	N60-N64	0.12	0.07	0.04	0.00	0.09	0.00	0.00	0.07	0.04	0.01
134	Inflammatory diseases of the female pelvic organs	N70-N77	0.16	0.06	0.09	0.00	0.12	0.00	0.00	0.10	0.05	0.01
135	Endometriosis	N80	0.17	0.11	0.06	0.00	0.13	0.00	0.00	0.10	0.06	0.00
136	Female genitals prolapse	N81	0.07	0.04	0.03	0.00	0.06	0.00	0.00	0.01	0.02	0.04
137	**15. Pregnancy and childbirth**	O00-O99	3.03	1.18	1.85	0.00	2.42	0.00	0.00	3.00	0.01	0.00

138	Ectopic pregnancy	O00	0.22	0.11	0.11	0.00	0.17	0.00	0.00	0.21	0.00	0.00
139	Medical miscarriage	O04	0.10	0.04	0.06	0.00	0.08	0.00	0.00	0.10	0.00	0.00
140	Hypertension in pregnancy	O10-O16	0.09	0.05	0.04	0.00	0.07	0.00	0.00	0.09	0.00	0.00
141	Placenta previa, placenta previa and antepartum hemorrhage	O44-O46	0.07	0.04	0.03	0.00	0.06	0.00	0.00	0.07	0.00	0.00
142	Obstructed labor	O64-O66	0.09	0.03	0.06	0.00	0.07	0.00	0.00	0.09	0.00	0.00
143	Perineal and vaginal lacerations during labor	O70, O71.4	0.10	0.04	0.06	0.00	0.08	0.00	0.00	0.10	0.00	0.00
144	Postpartum hemorrhage	O72	0.06	0.03	0.03	0.00	0.05	0.00	0.00	0.06	0.00	0.00
145	Normal labor and delivery	O80	0.33	0.07	0.26	0.00	0.27	0.00	0.00	0.33	0.00	0.00
146	**16. Perinatal diseases**	P00-P96	0.85	0.37	0.48	0.40	0.45	0.85	0.00	0.00	0.00	0.00
147	Birth injury	P10-P15	0.00	0.00	0.00	0.00	0.00	0.00	0.00	0.00	0.00	0.00
148	Birth asphyxia	P21	0.05	0.02	0.03	0.02	0.02	0.05	0.00	0.00	0.00	0.00
149	Neonatal aspiration syndrome	P24	0.05	0.01	0.03	0.02	0.03	0.05	0.00	0.00	0.00	0.00
150	Perinatal infections	P35-P39	0.05	0.02	0.02	0.02	0.03	0.05	0.00	0.00	0.00	0.00
151	Hemolytic disorders of the fetus and newborn	P55	0.02	0.01	0.01	0.01	0.01	0.02	0.00	0.00	0.00	0.00
152	Neonatal sclerosis	P83	0.00	0.00	0.00	0.00	0.00	0.00	0.00	0.00	0.00	0.00
153	**17. Congenital malformations**	Q00-Q99	0.48	0.36	0.12	0.22	0.26	0.14	0.09	0.12	0.08	0.06
154	Other congenital malformations of the nervous system	Q00-Q04，Q06-Q07	0.01	0.01	0.01	0.01	0.01	0.01	0.00	0.00	0.00	0.00
155	Congenital malformations of the circulatory system	Q20-Q28	0.22	0.18	0.05	0.10	0.12	0.04	0.02	0.06	0.06	0.05
156	Congenital heart disease	Q20-Q28	0.17	0.14	0.04	0.08	0.09	0.03	0.01	0.04	0.05	0.04
157	Cleft lip and cleft palate	Q20-Q28	0.01	0.00	0.00	0.00	0.00	0.00	0.00	0.00	0.00	0.00

158	Congenital malformations of the digestive system	Q38-Q40，Q42-Q45	0.03	0.02	0.01	0.01	0.02	0.02	0.00	0.00	0.00	0.00
159	Genitourinary Congenital Malformations	Q50-QQ52 , Q54-Q56	0.07	0.05	0.02	0.03	0.04	0.02	0.02	0.01	0.01	0.00
160	Congenital malformations of the musculoskeletal system	Q67-Q79	0.06	0.04	0.01	0.03	0.03	0.03	0.01	0.01	0.01	0.00
161	**18. Symptoms, signs and test abnormalities**	R00-R99	0.85	0.40	0.45	0.40	0.46	0.09	0.04	0.15	0.21	0.36
162	**19. Injury and poisoning**	S00-T98	6.58	2.46	4.12	3.06	3.51	0.24	0.33	2.09	1.97	1.95
163	Fractures	S02-S92, T02-T12	0.74	0.28	0.46	0.34	0.39	0.02	0.05	0.22	0.22	0.22
164	Skull and facet fractures	S02	0.13	0.05	0.07	0.06	0.07	0.01	0.01	0.06	0.03	0.02
165	Femur fractures	S72	0.95	0.40	0.55	0.44	0.51	0.02	0.02	0.08	0.12	0.71
166	Multiple fractures	T02	0.04	0.02	0.02	0.02	0.02	0.00	0.00	0.01	0.01	0.01
167	Intracranial injuries	S06	0.78	0.27	0.51	0.36	0.42	0.03	0.04	0.23	0.23	0.25
168	Burns and corrosive injuries	T20-T32	0.12	0.06	0.06	0.06	0.07	0.04	0.01	0.03	0.03	0.02
169	Poisoning by drugs, pharmaceuticals and biologicals	T36-T50	0.03	0.01	0.02	0.01	0.02	0.01	0.00	0.01	0.01	0.01
170	Toxic effects of non-medicinal substances	T51-T65	0.13	0.04	0.09	0.06	0.07	0.01	0.01	0.04	0.03	0.04
171	Medical complications	T80	0.14	0.09	0.06	0.07	0.08	0.00	0.00	0.04	0.04	0.05
172	Surgical and operative complications	T81	0.06	0.03	0.02	0.03	0.03	0.00	0.00	0.02	0.02	0.02
173	Prosthetic devices, implants and grafts complications	T82-T85	0.07	0.05	0.02	0.03	0.04	0.00	0.00	0.02	0.02	0.03

174	**20. Other medical services**	Z00-Z99	5.61	4.09	1.52	2.61	3.00	0.12	0.09	1.13	2.02	2.26

Note: 1) The value “0.00” is mainly used to indicate a high-precision approximation of the calculation results, and does not imply that the actual value is zero;

2) Due to rounding, there may be minor discrepancies between the sums of the sub-items and the totals in the table.
