# Supplementary figures and images for: Carbon footprint of the Chinese healthcare service: An environmentally extended input–output analysis
Source: PLoS Med. 2025 Sep 24;22(9):e1004738. doi: 10.1371/journal.pmed.1004738 (PMC12459823; doi:10.1371/journal.pmed.1004738)

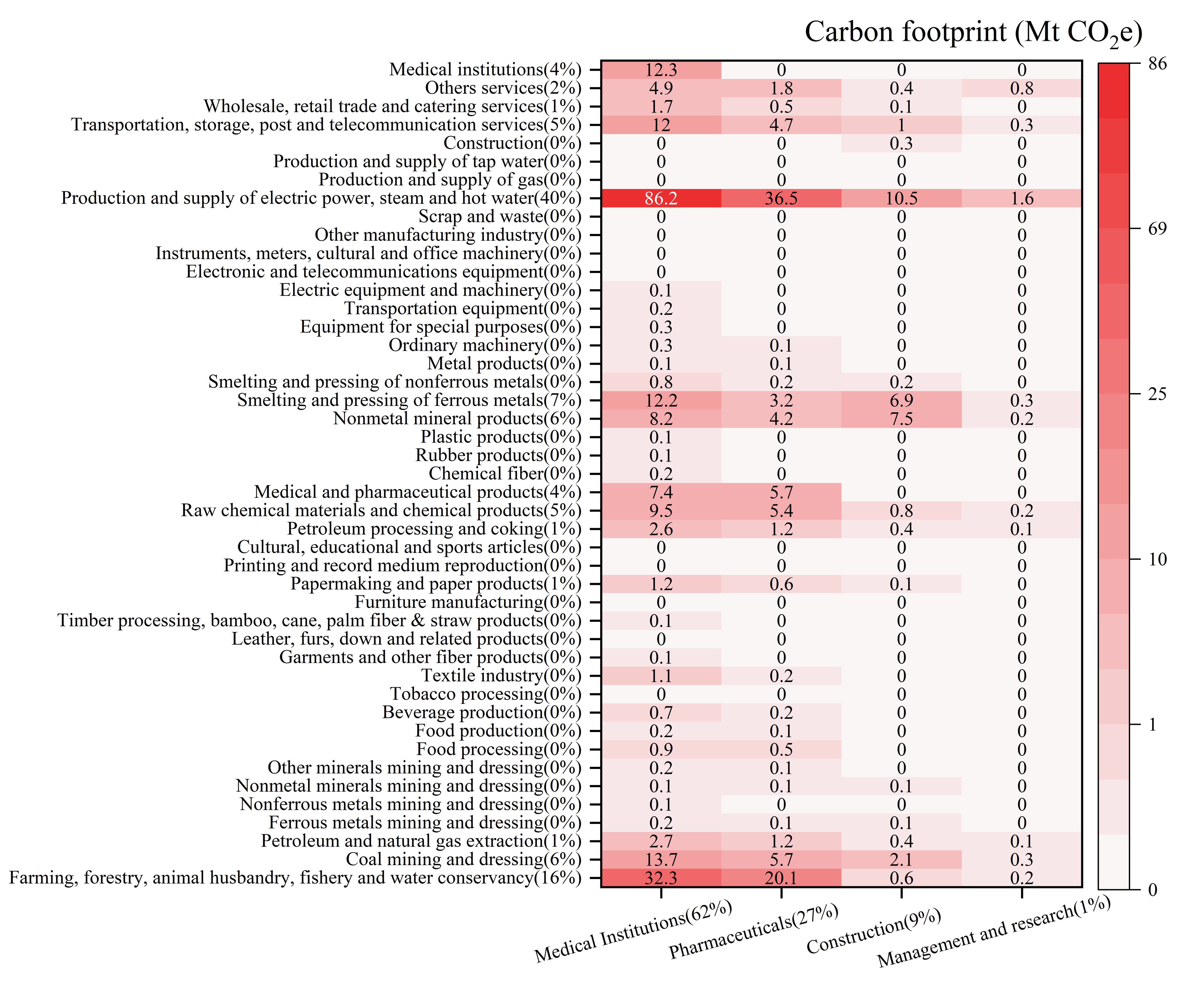

Supplement: S1 Fig — (TIF) [file pmed.1004738.s003.tif]

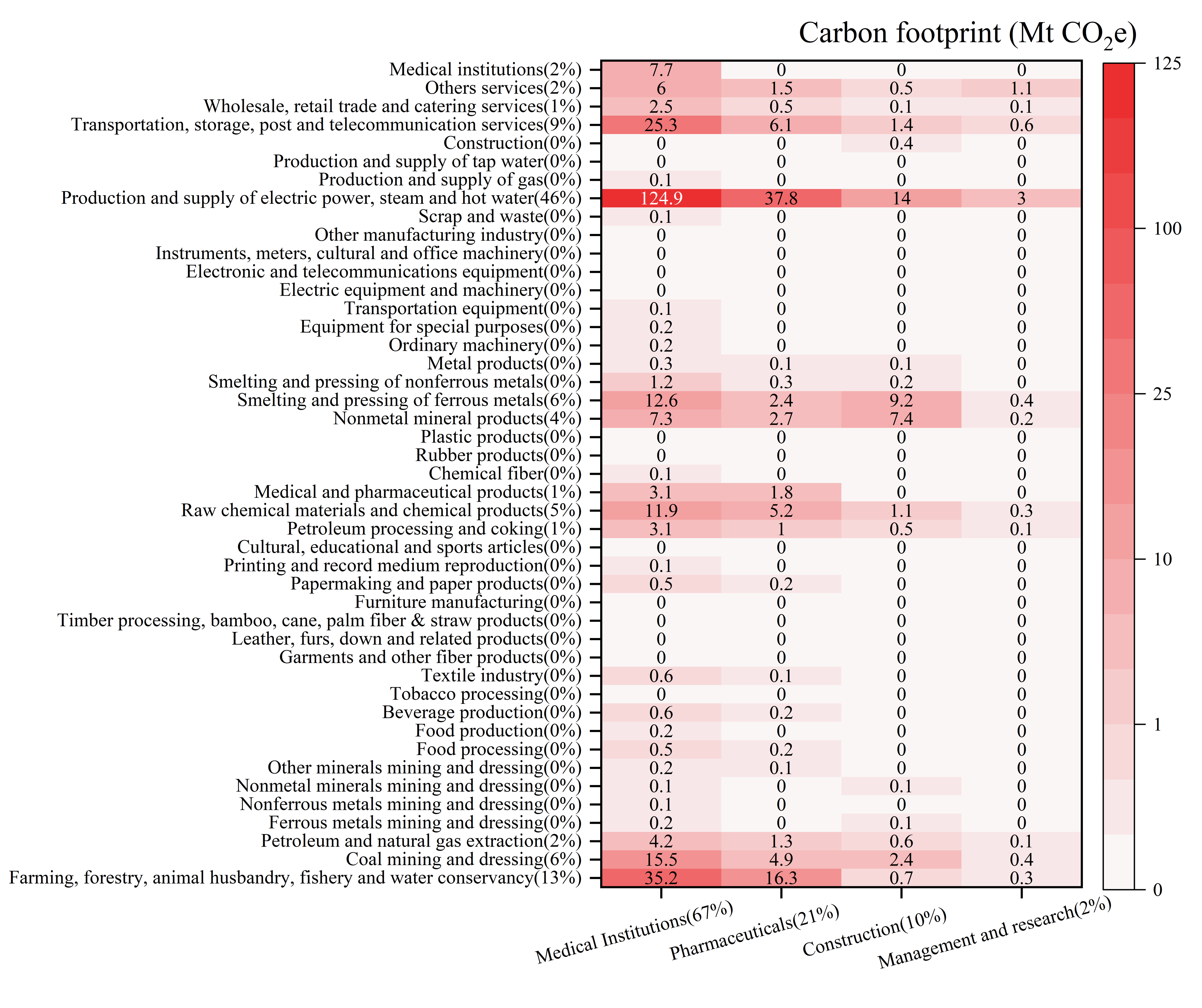

Supplement: S2 Fig — (TIF) [file pmed.1004738.s004.tif]

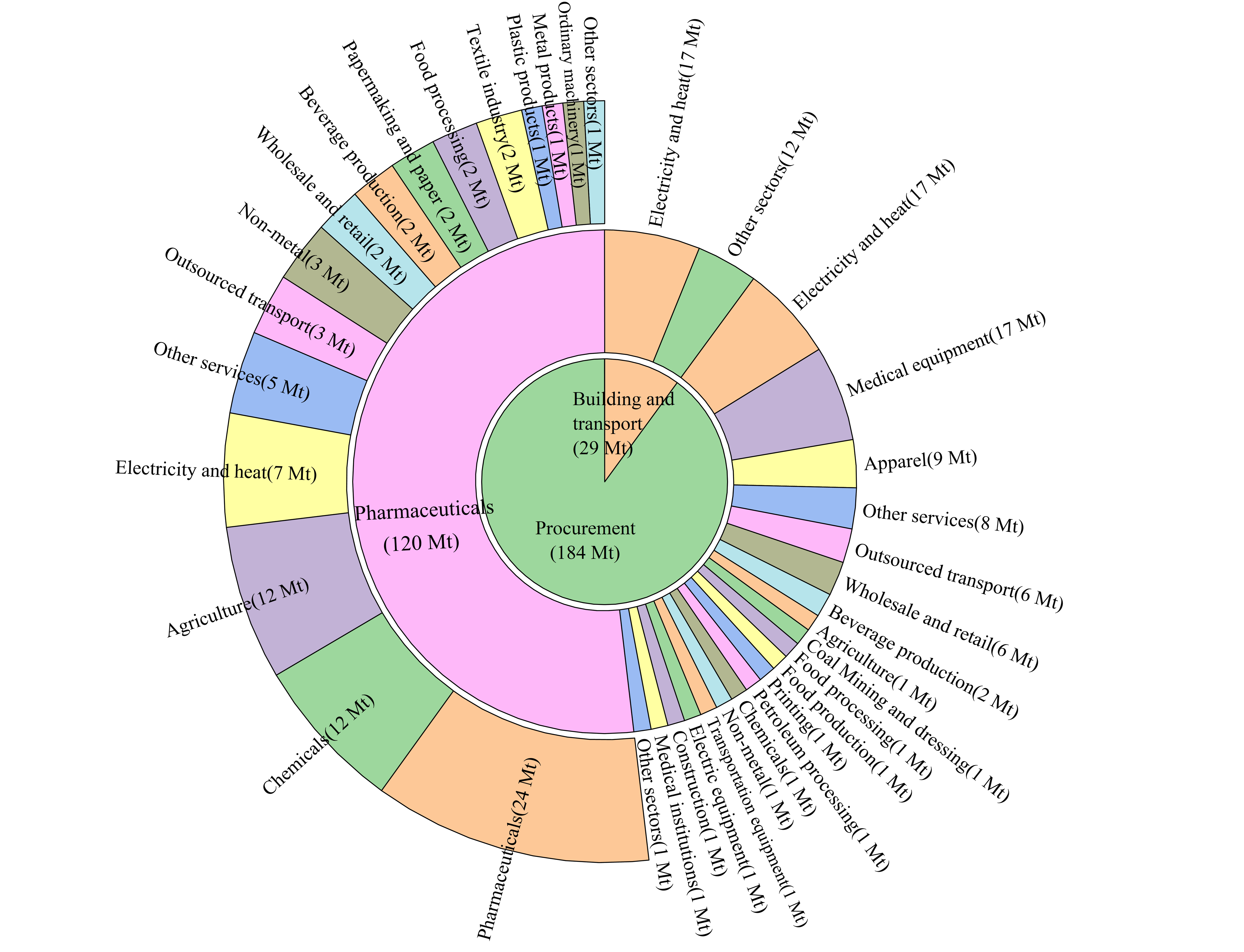

Supplement: S3 Fig — (TIF) [file pmed.1004738.s005.tif]

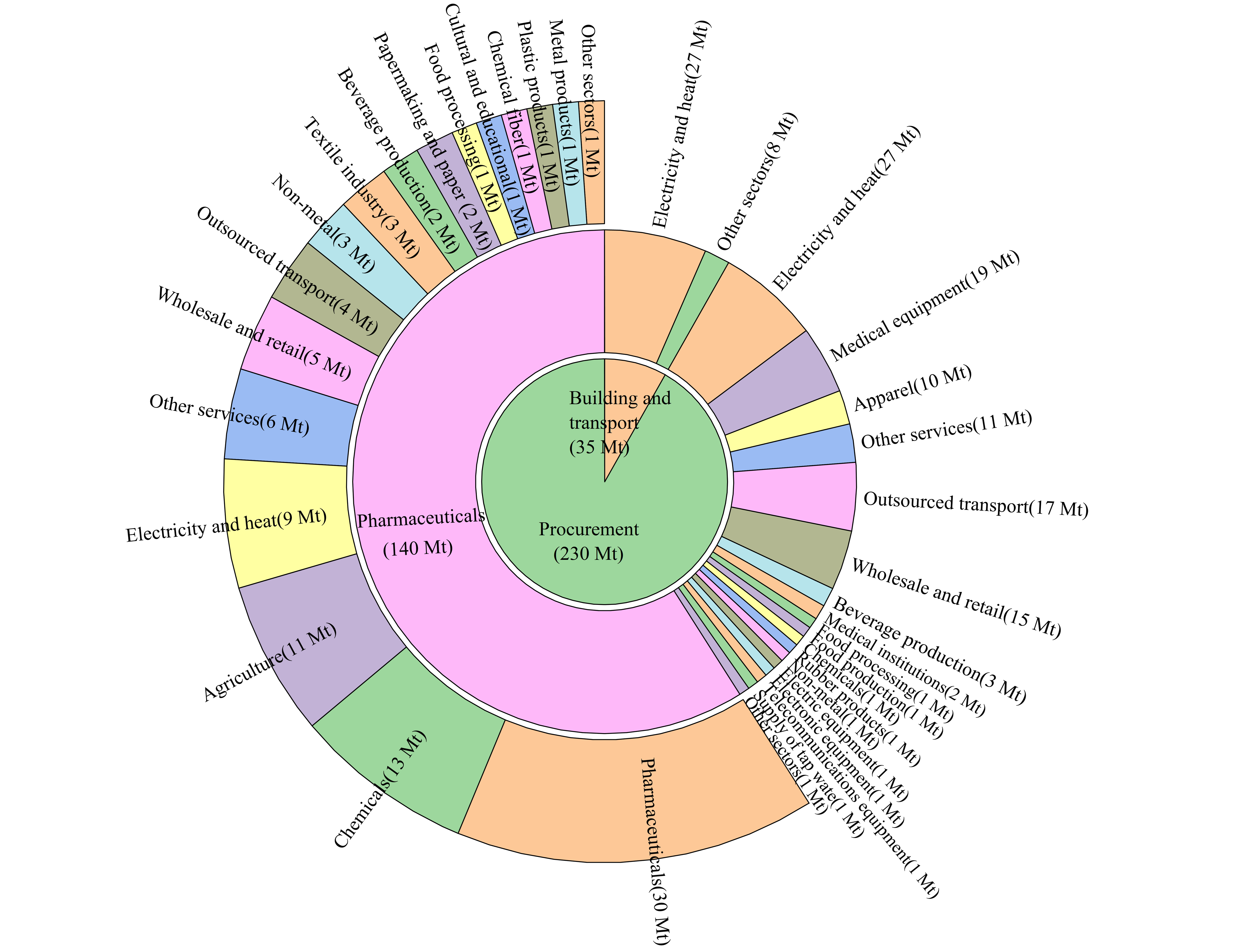

Supplement: S4 Fig — (TIF) [file pmed.1004738.s006.tif]
